# Therapeutic Strategies for Mutant *SPAST*-Based Hereditary Spastic Paraplegia

**DOI:** 10.3390/brainsci11081081

**Published:** 2021-08-18

**Authors:** Neha Mohan, Liang Qiang, Gerardo Morfini, Peter W. Baas

**Affiliations:** 1 Department of Neurobiology and Anatomy, Drexel University College of Medicine, Philadelphia, PA 19422, USA; nm3242@drexel.edu (N.M.); lq24@drexel.edu (L.Q.); 2 Department of Anatomy and Cell Biology, University of Illinois at Chicago, Chicago, IL 60612, USA; gmorfini@uic.edu

**Keywords:** Hereditary Spastic Paraplegia, spastin, *SPAST*, microtubule, *SPG4*-HSP, haploinsufficiency, loss-of-function, gain-of-function, gene therapy, casein kinase 2, HDAC6, autophagy

## Abstract

Mutations of the *SPAST* gene that encodes the microtubule-severing enzyme called spastin are the chief cause of Hereditary Spastic Paraplegia. Growing evidence indicates that pathogenic mutations functionally compromise the spastin protein and endow it with toxic gain-of-function properties. With each of these two factors potentially relevant to disease etiology, the present article discusses possible therapeutic strategies that may ameliorate symptoms in patients suffering from *SPAST*-based Hereditary Spastic Paraplegia, which is usually termed *SPG4*-HSP.

## 1. Introduction

Mutations of the *SPAST* gene (also called *SPG4*) account for the plurality of cases of autosomal dominant Hereditary Spastic Paraplegia (HSP), a heritable neurodegenerative disease involving progressive weakness and spasticity of lower limbs [1]. HSP patients display a characteristic gait deficiency, which eventually confines most of them to a wheelchair. *SPG4*-HSP is usually but not always adult-onset, with symptoms mainly resulting from degeneration of the corticospinal tracts. Corticospinal degeneration is characterized by pathological axonal swellings and dieback (distal to proximal) degeneration of axons and associated synapses [2].

**Figure 1:**
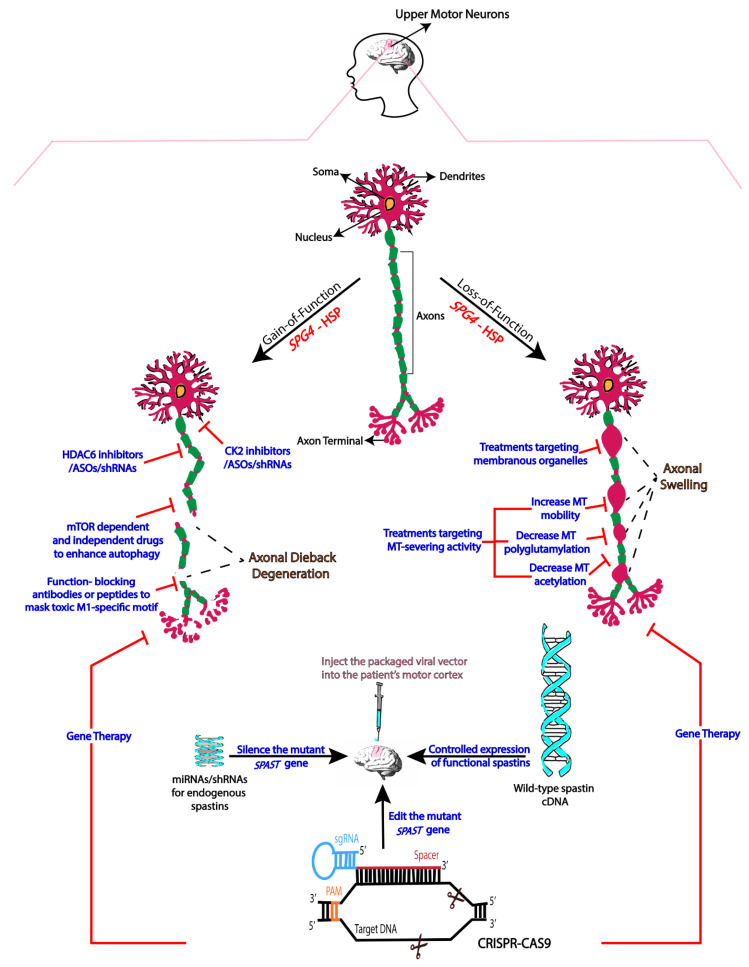
**Therapeutic approaches for targeting mechanistic components of *SPG4*-HSP.** Schematically displayed in the figure are gain-of-function and loss-of-function components of *SPG4*-HSP, and various potential treatment interventions discussed in this article that might target a specific component. Microtubule (MT).

*SPAST* encodes spastin, which is a microtubule-severing enzyme that also has functions relevant to the organization of membranous organelles [3,4]. An early idea was that insufficient levels of functional spastin account for the degeneration of the corticospinal axons [5,6,7,8,9,10,11,12], but more recent studies suggest that the gain-of-function toxicity of the mutant spastin is the primary culprit [13,14,15]. We have posited that both contribute, with loss of spastin function rendering axons more vulnerable to the toxic effects of the mutant protein [15]. If this is correct, strategies that treat either one (as summarized in Figure 1) may be useful in staving off the symptoms of the disease. Here, we speculate on different therapeutic approaches that may prove beneficial to patients with *SPG4*-HSP.

## 2. Mechanistic Basis of *SPG4*-HSP

A loss-of-function (i.e., haploinsufficiency) etiology for the disease was originally proposed on the basis of the genetics, but also on reports that failed to detect mutant spastin proteins in cells and tissues from human patients outside of the central nervous system [16,17]. The same disease apparently results from different kinds of mutations, including missense, nonsense, frameshift and truncation [18], which seemed consistent with haploinsufficiency. However, the cell biological features of the disease are not readily explicable on the basis of reduced spastin functions, with such a mechanism also failing to account for why no fetal abnormalities are apparent or why degeneration is mostly confined to the corticospinal tracts. In addition, *Spast* knockout mice with reduced spastin levels do not recapitulate most features of the disease [19,20], whereas mice expressing human mutant spastin display adult-onset gait deficiency and corticospinal dieback degeneration quite similar to the human patients [15]. Curiously, however, mice with reduced spastin display axonal swellings reminiscent of the human patients, whereas mice expressing human mutant spastin do not. These observations led us to wonder if loss-of-function and gain-of-function mechanisms might each have a role to play in the disease.

Transcripts produced from the *SPAST* gene have two start codons that produce a longer and a shorter isoform called M1 and M87, respectively [21,22]. In mice, the shorter isoform is termed M85 rather than M87 (due to sequence differences between human and mouse), but for simplicity we will refer to the shorter isoform as M87, regardless of species. M87 is widely distributed, while M1 is only detectably present in the adult cortex and spinal cord, where the corticospinal tracts reside [23]. Studies using a variety of experimental systems have consistently shown that mutant M1 protein is far more toxic than its M87 counterpart bearing the same mutation [13,14,23]. Mutant M1 protein also tends to be more stable than mutant M87, with the latter potentially becoming even less stable than its wild-type counterpart; this might cause mutant M1 to accumulate in the corticospinal tracts despite no mutant spastin protein being detectable in non-neuronal cells [23,24]. The idea that mutant M1 is more prone to misfolding than mutant M87 is consistent with the fact that the M1-specific domain, not present in M87, is extremely hydrophobic. Whether mutant M1 proteins form aggregates in afflicted neurons of *SPG4*-HSP patients remains to be explored.

We have reported experimental data suggesting that while the gain-of-function component drives the *SPG4*-HSP phenotype, the loss-of-function component exacerbates the gain-of-function component [3,15]. Given that some patients normally live for many decades with no symptoms, treatment of the loss-of-function component might be sufficient to provide other patients with extra years or decades of symptom-free life or might attenuate the symptoms to a more tolerable level. Thus, further exploration is merited to ascertain whether reduced spastin levels render axons more vulnerable to the toxicity of mutant M1, and if so, by what mechanism. However, if our logic is correct, amelioration of the toxic effects of mutant M1 is more likely to be therapeutically effective.

## 3. Therapies Based on Gain-of-Function Mechanisms

A common gain-of-function pathway for many neurodegenerative diseases is the aberrant activation of kinases and associated inhibition of fast axonal transport (FAT) by neuropathogenic proteins [14,25,26,27]. We have shown that mutant forms of M1 cause aberrant activation of casein kinase 2 (CK2), an effect not shared with their mutant M87 counterparts [14]. In squid axoplasm, pharmacological inhibitors of CK2, but not inhibitors of other kinases, rectify FAT deficits caused by mutant forms of M1, and the same is true in cultured neurons [14]. In fibroblasts, neuronal cell lines and primary cultured neurons, mutant forms of M1 cause organelle distribution defects and these too are corrected by CK2 inhibitors [14]. Specific subunits of conventional kinesin and cytoplasmic dynein, motor proteins directly involved in the execution of FAT, display aberrant patterns of phosphorylation in M1- but not M87-expressing cells, consistent with the notion that aberrant CK2 activation underlies the FAT deficits elicited by M1 in the squid axoplasm model [28]. A wide variety of other CK2 substrates, such as tau, neurofilaments and downstream kinases, may also be affected, any or all of which may contribute to degeneration [29]. An important step not yet taken is to ascertain whether restoring CK2 activity to normal levels could prevent degeneration of corticospinal axons and ameliorate the gait deficiency in the mouse model. If it can, then such an approach may be amenable to human patients, but not without concerns. A major obstacle is restoring the activity of kinases to normal levels without compromising other critical functions. Small chemical compound-based inhibitors or modulators are available for various kinases, but they all have off-target effects that may preclude their utility in patients, especially if higher dosages are required for the treatment. RNA interference or antisense oligonucleotides targeting a specific kinase may mitigate such concerns because they provide better specificity. Another concern arises from the multitude of substrates phosphorylated by most kinases, including CK2, whose activities impact many different cellular functions.

We have observed in different experimental systems that mutant M1 causes a decrease in microtubule acetylation as well as a decrease in microtubule stability [24]. It may be that the former causes the latter, which is consistent with the fact that changes in microtubule acetylation cause changes in association of the microtubules with proteins that can regulate its stability properties [30]. Simpler yet would be if the latter causes the former, given that microtubules that are less stable are known to accumulate fewer acetylated subunits. This makes sense as a potential explanation for the FAT defects caused by mutant M1 because conventional kinesin (Kinesin-1), the predominant motor for anterograde FAT, reportedly interacts better with microtubules that are richer in acetylated tubulin [14,24]. While this needs to be explored further, there are many preclinical studies on models of other neurodegenerative disorders in which the pharmacologic inhibition of HDAC6, the chief tubulin deacetylase [31], restores deficits in microtubule acetylation and thereby rectifies FAT deficits as well as behavioral and anatomical deficits [32,33]. Alternatively, microtubule-stabilizing drugs have been suggested to correct the situation, with some supportive evidence at the cellular level [34]. Noscapine, a mild microtubule-stabilizing drug used as cough medicine in several parts of the world, is being considered for clinical trials for *SPG4*-HSP in Australia, with pre-clinical studies ongoing [35,36].

It remains to be seen whether the correction of either of these two gain-of-function effects ameliorates and/or prevents the behavioral deficits of *SPG4*-HSP in humans or animal models. As previously done for other mouse models [37], genetic experiments in mutant spastin mice with fluorescently labelled corticospinal motor neurons could help determine the extent to which cell-type specificity contributes to the degeneration of corticospinal tracts. While such experiments and trials are in the works, another approach worth considering is to enhance the capacity of the axon to eliminate the toxic mutant spastin protein through the pharmacological enhancement of autophagy. In fact, mutant M1′s negative effects on FAT may provide a positive feedback loop by reducing the efficacy of autophagy such that restoring autophagy to robust levels may be effective therapeutically. The enhancement of autophagy to rid the axon of misfolded toxic proteins is an active area of research for many neurodegenerative disorders [38,39,40,41,42], and hence progress on other diseases may provide a path forward for *SPG4*-HSP patients. Rapamycin and other drugs targeting the autophagy activator mTOR are being tested in preclinical studies of other diseases, but effects of these drugs go beyond autophagy enhancement, so the results must be interpreted with caution. Finally, another therapeutic strategy targeting gain-of-function mechanisms could involve the development of peptides or antibodies targeting specific regions of mutant M1 spastin conferring toxicity. The precise mapping of such regions is an active line of research in our laboratories.

## 4. Therapies Based on Loss-of-Function

There is evidence that spastin dosage is crucial for the health of the axon, at least when confronted with challenges such as regeneration after injury [43]. Therefore, it is no surprise that a reduction in the levels of active spastin might render the axon more vulnerable to the toxicity of mutant M1 [3]. If this is the case, what functions of spastin are at the heart of this, and what can be done therapeutically to compensate for the ill effects of reduced spastin activity? One possibility is that the vulnerability has less to do with spastin’s microtubule-severing properties and more to do with functions in the organization of cellular organelles such as endoplasmic reticulum, endosomes and mitochondria [44,45,46,47,48]. Here, we will consider therapies based on reduced microtubule severing because they are more therapeutically tractable.

Spastin preferentially severs axonal microtubules in the stable domain of the microtubule, as opposed to the labile/dynamic domain [49]. This reduces the total proportion of the microtubule mass that is stable and acetylated and creates numerous short stable microtubules that are highly mobile and able to elongate, thus increasing microtubule number. Reductions in microtubule severing result in a microtubule array that is more stable and more acetylated, with a smaller number of longer microtubules [43]. There is also less mobility within the microtubule array because microtubule mobility is mainly confined to very short microtubules. What is curious about these effects, which have been experimentally demonstrated [15], is that they are partly the opposite of the gain-of-function effects on microtubules discussed above. It will be interesting to explore mouse and cellular models that simultaneously depict the loss-of-function and gain-of-function components of the disease, in order to ascertain whether the lowered or heightened microtubule stabilization/acetylation predominates, if they cancel one another out, or if they occur sequentially. Potentially important is that acetylation itself is not the tubulin modification that actually targets spastin to the stable domain of the microtubule; rather, the relevant modification is polyglutamylation [50,51], and hence exploration into its regulating enzymes and cell biological consequences may be useful toward devising therapies for *SPG4*-HSP [52,53].

It may be that reduced microtubule mobility underlies the vulnerability of corticospinal axons associated with lower spastin microtubule-severing activity. Reduced microtubule mobility imposes a burden on the axon because fewer tubulin subunits are supplied for the proper maintenance of the MT array and because microtubule mobility is the primary means by which incorrectly oriented microtubules are cleared from the axon [54]. Microtubules in the axon are almost entirely oriented with plus-end-out, with flipped microtubules moving back into the cell body to preserve the axon’s microtubule polarity pattern from corruption. The accumulation of microtubule polarity flaws (i.e., flipped microtubules) is an appealing mechanistic explanation for the appearance of axonal swellings because such swellings can be caused by “traffic jams” of vesicular organelles undergoing FAT. Local regions of mixed microtubule polarity orientation could explain such traffic jams. Another possibility is that microtubule polarity flaws are caused not by diminished microtubule mobility but by increased microtubule acetylation causing aberrant directionality of microtubule movements due to enhanced Kinesin-1 interaction with the microtubules [55]. Cytoplasmic dynein is the motor protein responsible for “polarity sorting” the microtubules in the axon [54,56], and abnormal microtubule movements by aberrantly increased Kinesin-1 activity would cause problems for that mechanism. More work needs to be done to test these ideas, but clarity on these issues may point the way toward therapies.

## 5. Gene Therapy

Gene therapy is achieving success in the treatment of genetic diseases. For example, the FDA has approved innovative gene therapy to treat pediatric patients with spinal muscular atrophy, a rare neurological disorder and the leading genetic cause of infant mortality [57]. One idea for *SPG4*-HSP would be to introduce into the patient’s motor cortex a viral vector that includes micro-RNA (miRNA) or short hairpin RNA (shRNA) to deplete the patient’s endogenous spastin, as well as the cDNA that expresses wild-type spastin under its endogenous promoter. Theoretically, any loss-of-function and gain-of-function defects would thereby be resolved. However, this strategy is not without potential snags. The level of spastin expression cannot be too high because too much spastin could tear down the microtubules of the cells to a detrimental level. Because the mutant M1 already accumulated in the corticospinal tracts may be long-lived, there is uncertainty as to how long it would take for that mutant M1 to be degraded after turning off the expression of new mutant protein. There may be a window of time past which this strategy may stop degeneration and promote regeneration, but to the detriment of patients should regenerating axons become misdirected. Additionally, there are potential issues of non-specificity or other unexpected side effects in viral-driven gene therapy approaches. Another idea is to specifically target the mutant gene for silencing while allowing the wild-type gene to continue to express normally, with the hope that haploinsufficiency would be tolerable if the toxic mutant protein can be sufficiently reduced.

The most contemporary approach, which circumvents some of these potential snags, is gene editing via CRISPR-Cas9. This technology, which is currently undergoing testing for the treatment of other neurodegenerative disorders [58,59,60], directly corrects the mutation of the endogenous gene at the genomic level. While this is seemingly ideal, the approach nevertheless poses its own potential for off-target effects due to genome rearrangements [61,62,63]. These effects may be mitigated by a reduction in the level of interaction between Cas9 and DNA to allow for a stronger connection between the DNA/RNA complex to enable more accurate genomic edits [64].

The very best approach for treating patients with *SPG4*-HSP may be coupling gene therapy to one or more of the other approaches discussed earlier to ameliorate the ill effects of the accumulated mutant M1 due to its resistance to degradation. For these various reasons, it may be that gene therapy is best suited for staving off the corticospinal degeneration of patients who are early in the display of symptoms, or asymptomatic patients. The rare pediatric cases of the disease may also be especially amenable to gene therapy because the nervous system of a child is still plastic/developing, because not as much mutant M1 would have accumulated in a young person, and because autophagy machinery is relatively more active at younger ages.

## 6. Concluding Remarks

*SPG4*-HSP is an autosomal dominant monogenic disorder that is theoretically far more straightforward to understand mechanistically than multifactorial disorders such as Alzheimer’s disease. *SPG4*-HSP qualifies as a rare disorder and yet there are hundreds of thousands of affected patients worldwide. Developing effective therapies based on an understanding of disease etiology (see Figure 1) will not only help those millions of sufferers but will also likely provide a paradigm by which other diseases can be treated. Although *de novo* mutations do appear, most *SPG4*-HSP cases are hereditary and hence can be identified with genetic testing prior to the appearance of symptoms. Thus, treatment can begin early to stave off progression of the disease long before the symptoms become debilitating and irreversible. With a rapidly growing understanding of the disease and the development of powerful animal and experimental models, there is every reason to predict that effective therapeutics for *SPG4*-HSP are within our grasp. In the present article, we have outlined various avenues toward treatment that we hope to pursue either alone or in combination for the effective amelioration of the symptoms of the disease.
